# Tuberculosis and Experiments on Murderers

**Published:** 1881-01

**Authors:** 


Tuberculosis and Experiments on Murderers.—There have been established by repeated experiments a large number of facts regarding the infectiousness of tuberculosis. It is known that the disease may be communicated from man to lower animals, and from one of the lower animals to the other. The only point yet left undemonstrated is, whether the tuberculous virus from lower animals can, under any ordinary conditions, infect man. Yet this point is by far the most important one to be established. It is, indeed, the especial goal of all the work and study upon the subject. We have, on the one hand, the fact that one-fifth of all deaths are caused by pulmonary phthisis; with which disease tuberculosis has so close a connection, if not a nearly uniform identity. But besides this great mortality from consumption, there are thousands of deaths annually from tuberculous diseases of the intestines and meninges..

On the other hand there are three or four million head of cattle in the country. If tuberculous disease is as frequent here as in Germany, which we have reason to believe is the case, there must be a vast number of infected animals among our herds. Yet from them the people are being constantly supplied with milk and meat. The importance, therefore, of seeing that so immense a source of food-supply be a healthy one, is strikingly apparent.

We know of only one certain way by which the question whether man can be infected by the milk or flesh of tuberculous cattle can be settled. This is by making the experiment upon man himself—upon criminals condemned to death. There is nothing cruel or at all revolting in the idea. For a certain period previous to the day for execution, the person to be experimented on should be fed with the milk or flesh, or both, of tuberculous cattle. There need be nothing offensive in such a diet. The criminal's physical condition should be carefully watched to see whether tubercles develop. After execution, a careful necropsy should be held. By experiments properly conducted in this way we should secure results of the highest importance to science, and preventive medicine.

